# Experiences of language barriers by homoeopathy student interns providing health services at the University of Johannesburg

**DOI:** 10.4102/hsag.v26i0.1491

**Published:** 2021-03-29

**Authors:** Lorna Blackwell, Neil T. Gower, Reshma Patel

**Affiliations:** 1 Department of Complementary Medicine, Faculty of Health Sciences, University of Johannesburg, Johannesburg, South Africa

**Keywords:** communication, healthcare delivery, healthcare process, healthcare provider, homoeopathy, health services, interpreter, language barriers

## Abstract

**Background:**

South Africa is a multilingual society, and therefore, the likelihood of healthcare providers (HCPs), including students training to be HCPs, encountering language barriers with patients is high.

**Aim:**

To explore and describe the experiences of homoeopathy student interns regarding language barriers in the delivery of health services and to provide guidance towards overcoming language barriers in homoeopathic practice at the University of Johannesburg (UJ).

**Setting:**

The interviews and focus group discussions were conducted in English and at a private location that was convenient for the participants in Johannesburg, Gauteng.

**Methods:**

This qualitative study used a phenomenological approach. Ten individual interviews were conducted with registered homoeopathy student interns (HSIs) from the UJ. The central question: ‘What has your experience been regarding language barriers between you and your patients at the UJ Homoeopathy clinics?’ was asked and responses were recorded and transcribed for later analysis. The interview results were presented to a focus group for discussion to validate findings that arose from the analysis and to provide an opportunity to add any insight, comment or recommendations that were not expressed in individual interviews and verification of emergent themes.

**Results:**

Participants described how language barriers create challenges in understanding between patients and HSIs. Descriptions of the experiences of the intrapersonal and interpersonal effects that are associated with language barriers were provided. Participants also described the influence of language barriers on the various aspects of the health service process. Finally, participants described the mitigation of language barriers through various strategies.

**Conclusion:**

Participants reported their experiences of language barriers as challenging. Language barriers were found to adversely affect the various aspects of the health service process as well as the practitioner’s personal feelings and the patient–practitioner relationship. Language acquisition and awareness modules introduced early on in the syllabus is a proposed solution to the mitigation of language barriers.

## Introduction

Patient diversity is influenced by various factors including race, sex, gender, culture, socioeconomic status and, not least of all, language. With 11 official languages spoken in South Africa, healthcare providers (HCPs) will routinely encounter a diverse patient base, if based only on language spoken. Benjamin et al. (2016) stated that a reality for South African society is that the majority of health consultations (as much as 80%) are not performed in the home language of the patient and has described the interplay of language and health as ‘a monolingual health service in a multilingual society’.

The pre-eminent position of English in the healthcare field is problematic as detailed and accurate communication and understanding during case management is vital (Paternotte et al. 2015). Parsons et al. (2014) and Zendedel et al. (2016) argued that language barriers and miscommunication have a negative effect on the health service process. Language barriers in healthcare may result in misunderstandings during case management as well as legal and ethical implications when making use of informal interpreters (Engelbrecht et al. 2008). In contrast, patient-centred communication allows the practitioner and patient to make decisions regarding the patient’s health together; however, language barriers block this process from occurring (Paternotte et al. 2015).

Systematic reviews conducted by Paternotte et al. (2015) and Ahmed et al. (2017) concluded that language barriers proved challenging to both patients and doctors and recommend that solutions in training HCPs for effective communication are needed. Two other studies in which individual interviews were conducted on HCPs showed that doctors need more support and education in handling language barriers as they felt their lack of skill showed incompetence and an inability to accurately judge if information collected under these circumstances was sufficient (Parsons et al. 2014; Skjeggestad et al. 2017).

A South African study demonstrated that doctors are unsatisfied with the language barriers they encounter and wish to learn their patients’ languages whilst the implementation of professional interpreter services in hospitals negates the language barriers experienced (Deumert 2010). Professional interpreter services and language courses relevant to the particular region have been recommended to be implemented for HCPs (Engelbrecht et al. 2008).

Whilst such studies investigating language barriers in the field of health service delivery have been conducted both nationally and internationally, no studies are known to have investigated the experience of language barriers amongst those who practice complementary medicine, such as homoeopathic practitioners.

In South Africa, homoeopathic practitioners, registered with the Allied Health Professions Council of South Africa (AHPCSA) and registered students in training for this profession, providing health services fall under the category of HCPs (South Africa 2003). Homoeopathic practitioners and homoeopathy student interns (HSIs) should be prepared for dealing with situations where a language barrier is encountered in order to ensure the provision of high-quality services. As a detailed oral description of their health status by patients is central to the medical and therapeutic method of homoeopathic professionals, the lack of precise communication may hinder quality outcomes.

Whilst the 5-year coursework prepares homoeopathy students to fulfil their future scope as practitioners, there is no significant training in African languages or cultures offered in any modules despite the diverse demographic of patients visiting the Homoeopathy Health Centre at the University of Johannesburg (UJ) (Kara 2019). This potentially may leave students unprepared in dealing with a diverse patient base. Helping the students to become more prepared in this regard may benefit both parties involved in the delivery of health services. Understanding the experiences of the students who encounter these patients with language barriers may help to provide some guidance in this regard.

## Research purpose

The purpose of this study was to explore and describe the experiences of HSIs regarding language barriers in the delivery of health services and to provide guidance towards overcoming language barriers in homoeopathic practice at the UJ.

## Research objectives

The following research objectives are outlined:

- To explore and describe HSIs experiences of language barriers in the delivery of health services by conducting individual interviews
- To determine what measures have been employed by HSIs in order to overcome the language barriers they encounter
- To describe the communication between HSIs and patients with diverse language backgrounds

## Definitions and descriptions of key concepts

Understanding the following key concepts is important as they are relevant for this study:

### Language barrier

In the context of this study, a language barrier constitutes an instance whereby the student cannot communicate with a patient because of differences in languages spoken or differences in proficiencies of a common language.

### University of Johannesburg health training centres

There are three training facilities where HSIs from the UJ conduct consultations with patients, including UJ Health Centre on the Doornfontein campus, the Soweto satellite services and the Ennerdale satellite services.

### Delivery of health services

In the context of this study, the delivery of health services refers to the services provided by the HSI to the patients they may interact with under supervision. The process of this health service involves conducting a consultation with the patient, performing relevant physical examinations, the conduct of special investigations, arriving at a diagnosis, and selecting a prescription and treatment plan.

### Interpreter (versus translator)

An interpreter is a person who translates between two languages in an oral context; a translator is a person who translates between two languages in a written context (Refugee Health Technical Assistance Center 2011). Participants in this study often used the two interchangeably, but in all cases, the concept of an interpreter, rather than a translator, was implied.

## Materials and methods

### Design

This study used a qualitative design with a phenomenological approach. A qualitative method was most appropriate because of the exploratory nature of the topic, allowing the researcher to collect data that helped gain insight into the students’ experiences on language barriers. The use of a phenomenological approach allowed for exploration into the experiences of participants regarding a particular phenomenon, in this case, language barriers, resulting in a particular theory regarding that phenomenon (Bricki & Green 2007; Fawole 2014).

### Population

The research population most suited to this study were students registered with the AHPCSA (HSIs) who were involved in providing health services under supervision to patients at the UJ Health Training Centres.

### Sampling and participants

The participants for this research were recruited using a purposive sampling technique. From a possible research population of 37 HSIs, 15 HSIs who were actively engaged with clinical activities at the time of the study were made aware of the study and were requested to participate. All 15 HSIs volunteered to participate in the research study and were confirmed to be eligible to participate.

The sample size was determined by data saturation, which was after 10 interviews. The interviews were followed by a focus group consisting of six HSIs drawn from those who had volunteered for the study. Only one of the six focus group participants was part of the interview sample.

### Data collection procedures

Individual semi-structured interviews allowed for data to be collected from participants. During the interviews, participants were asked the central question: *What has your experience been regarding language barriers between you and your patients at the UJ Homoeopathy clinics?* and were encouraged to answer this central question as openly and honestly as possible. The researcher utilised a list of guiding questions to prompt any response or further description of answers where it was required:

- Can you give me examples (related to any stated experiences or perceptions)?
- What do you consider to be a language barrier?
- Do you think this affects your patient in any way?
- How do you think it (language barriers) makes the patient feel?
- Do you think this affects you as a doctor in any way? Or your process?
- How does it (language barriers) make you, as a doctor, feel?
- What do you do when you encounter these language barriers?
- What do you think would help remedy the situation?
- Have you ever used an interpreter before? What was the experience like?
- Is there anything you wish to add on the topic of your experience of language barriers?

Participants were also asked to fill in a short table listing their first language and any other languages they speak. They then had to rate their perceived proficiencies of the other languages as ‘below average’ (a few words), ‘average’ (conversational) or ‘above average’ (fluent) for demographic purposes. The interviews were conducted by the researcher in person at a location most convenient for the participant and were on average 30 min long. The interviews were electronically recorded in order to be transcribed at a later stage. Data saturation was reached after 10 interviews were conducted.

### Content analysis

The researcher interviewed and transcribed the interviews herself. The researcher then coded and recoded all 10 of the transcripts using the qualitative analysis software Atlas.ti version 8. Subsequent codes were organised into categories and then further into themes. The researcher presented the codes, categories, themes, memos and transcripts to an external qualitative analyst who was able to verify the process used by the researcher and provided input regarding themes where applicable. The researcher then presented these themes to a focus group at the UJ for discussion, verification and clarification. The focus group agreed with the themes presented, which enabled the researcher to proceed with an accurate representation of experiences.

### Ethical considerations

The study was approved by the UJ Faculty of Health Sciences Research Ethics Committee (REC-01-40-2018). Purpose of the study, procedures and use of information gathered was explained by the researcher and was contained in the documents provided to participants prior to the interviews. Participants who were willing to participate signed the relevant documents. All participants and their ideas and opinions were treated with respect.

Ethical considerations considered in this study included autonomy, confidentiality and privacy, and justice.

Autonomy was ensured by placing importance on the participant’s consent and understanding of the purpose of the interview and how the information that they provide was used. The procedure of the interview process was explained, and an information sheet was provided to participants before the interview commenced. The participant signed letters of consent – to an interview, to be recorded and to information use by the researcher – which were also explained to the participants and signed before the interview commenced.

To ensure privacy all interviews and the focus group discussions were conducted in a private room. Confidentiality was ensured by assigning each participant a code in order to identify the different transcripts after the researcher has reviewed the transcript. Furthermore, transcripts were saved on a device, which was password protected and was further saved to a secure cloud dedicated to this research, access to which was also password protected. Only the researcher had access to this information. This ensured the safety and privacy of the information obtained. All data will be destroyed 2 years after the publication of this dissertation.

All participants in this study were treated equally and respectfully. The participants were seen at times and locations that were at their convenience. Participants’ ideas and answers were respected. All participants have access to the results of this study. Participants were permitted to withdraw from the study at any point without repercussion.

### Measures of trustworthiness

As the findings of the study are based on the experiences and observations of the participants, trustworthiness is ensured through credibility, transferability, confirmability and dependability (DeMotts 2018).

Credibility was ensured in a variety of ways. Triangulation methods involving individual interviews, member checks in the form of a focus group and an extensive literature review of the phenomenon under scrutiny were performed. Participants were encouraged to share openly and honestly and given ample opportunity to express their opinions and experiences throughout the interview process. These interviews were recorded with two devices to ensure maximal clarity of sound data collected. Transcription was conducted by the researcher herself ensuring familiarity and accuracy. The researcher used dedicated qualitative analysis software (Atlas.ti 8) for which a 2-day intensive training workshop was attended to ensure the most appropriate and correct use of the program for analysis. The researcher recorded memos that detailed their own critical reflection throughout the data collection and analysis processes.

Transferability was facilitated by using a purposive sampling method. The participants were selected by the researcher as these individuals fit specific criteria that would allow the researcher to obtain the richest and most up-to-date information.

Dependability is ensured through detailed documentation of methodology employed by the researcher, as well as making use of various overlapping methods of data confirmation and collection, that is, conducting a pilot study, conducting individual interviews, as well as conducting focus group discussions.

Confirmability was ensured through the taking of field notes, which included a section for critical reflection and memos that were written during the coding process of data analysis. These field notes and memos allowed for the identification of common topics, improvement of techniques and rationale for choices made during the coding process. Bracketing was performed in the form of memo writing so as to allow reflection and avoid the researcher’s interpretations and personal beliefs about the phenomenon contaminating the true essence of what the participant was describing.

## Results

### Demographic profile of participants

**FIGURE 1:**
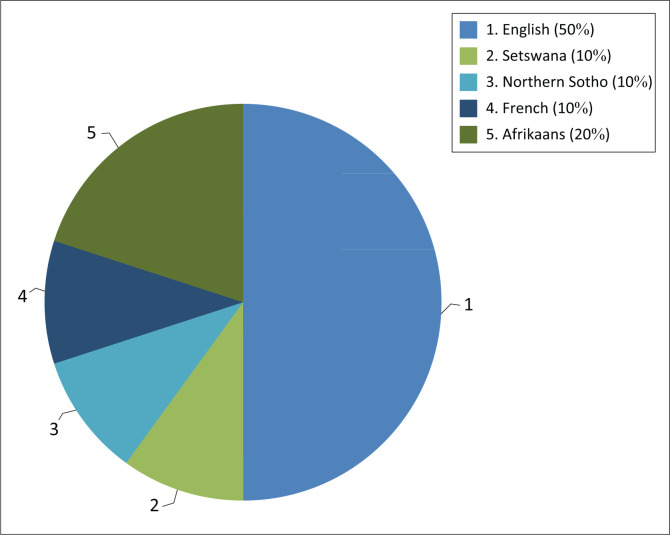
First languages spoken by participants.

A total of 10 participants were interviewed, comprising of two males and eight females. As per Figure 1, half (50%) of the sample reported English as their first language, and the remaining five participants (50%) reported English as an additional language.

All five English first language speakers listed Afrikaans as an additional language and one participant included Portuguese as an additional language. Of these five participants, only two listed isiZulu as an additional language and both rated their perceived proficiency of isiZulu as poor (only being able to speak a few words).

The home languages spoken by participants who listed English as an additional language included Afrikaans (two), French (one), Northern Sotho (one) and Setswana (one) (Figure 1).

Of the five participants who listed English as an additional language, four reported their English proficiency as being above average (fluent) using the perceived proficiency score chart provided. Only one participant rated his or her proficiency as average (conversational). None rated their perceived proficiency of English as poor (only knowing a few words).

Only two participants listed isiZulu as an additional language with poor proficiency. None of the participants listed their first language as isiZulu. In contrast, in Gauteng (the location of the study), isiZulu is the most spoken language (14%), whilst English is the second most spoken language (11%) (Statistics South Africa 2012).

### Interviews

From the content analysis, four themes emerged: (1) language barriers lead to a lack of understanding between the HSI and patient and are therefore challenging to deal with, (2) various aspects of the health service process are negatively influenced by the presence of language barriers, (3) the rapport between HSIs and patients, as well as intrapersonal communication is negatively affected and (4) attempts by HSIs to mitigate these negative effects are employed. Verbatim quotations from the transcripts are provided as examples to illustrate the identified themes.

### Theme one: Language barriers create challenges in understanding

Participants described understanding patients when there is a language barrier present between them as challenging. Participants described difficulty in understanding what the patient is trying to communicate or express, and therefore details are lost and inaccurate information risks are being recorded:

‘I felt that sometimes perhaps I didn’t understand the detail, so the detail got a little bit lost.’ (code 49.3)

‘Because it was very obvious in a sense that we were speaking of two completely different things.’ (code 20.10)

This is in accordance with many studies which show that language barriers have an effect on a multitude of aspects in healthcare including patient satisfaction, adherence to advice and medication, diagnosis, costs, mortality and HCP-patient relationships because of the misunderstandings that result (Ahmed et al. 2017; Deumert 2010; Almutairi 2015; Ashkinazy 2017; Levin 2006, 2011; Li et al. 2017; Lukoschek, Fazzari & Marantz 2003; Parsons et al. 2014; Taylor, Nicolle & Maguire 2013; Van den Berg 2016).

### Theme two: Intrapersonal and interpersonal effects associated with language barriers

Language barriers result in the students’ experiencing negative emotions during their interaction with the patient. Participants described feelings of frustration and inadequacy as they feel they are not good enough or are not doing as much as they could for their patients because of the language barriers present between them. Participants also described feelings of insecurity and uncertainty as they do not know whether or not they are doing the right thing, and they are not sure of how to proceed in the presence of the language barrier. Participants felt that if they were able to communicate without the barrier of language, professionalism, respect and care for the patient would be more easily attainable:

‘It’s very frustrating. I get frustrated.’ (code 13.2)

‘And anxious, I suppose, because what am I not getting from this patient that I should be getting. Yes, I’d be feeling insecure that I might be missing stuff.’ (code 15.7)

Paternotte et al. (2015) stated that doctors found it challenging to relate to patients because of perceived incompetence in communication skills, which would severely affect the doctor–patient relationship. Effective communication results in patients feeling more comfortable, more understood and results in higher rapport with their HCP. Patients also rate their care of a higher quality if they were able to communicate effectively. This may occur even when the HCP is unable to speak the patient’s language yet makes an effort to communicate in their language (Levin 2006; Paternotte et al. 2015).

### Theme three: Language barriers’ influence on the various aspects of the health service process

The health service process consists of a consultation, physical examination, resultant diagnosis, homoeopathic prescription and treatment plan. These steps are aimed at eliciting an adequate amount of relevant information that will guide the student through the process and appropriately diagnose, treat and manage a patient’s case. Homoeopaths tend to conduct longer consultations that align with what patients place in high regard – such as empathy, individualisation and the ability to express their concerns in-depth (Eyles, Leydon & Brien 2012).

Language barriers influence the various aspects of this health service process by causing a communication breakdown resulting in difficulty obtaining accurate and detailed information. Verghese and Ioannidis (2017) confirmed this by describing how the doctor may overlook, misinterpret or completely miss an important and valuable symptom if the patient is unable to communicate to the practitioner what they are experiencing:

‘So, I think it sort of disables your process because you aren’t able to gain as much detail as you would need.’ (code 49.4)

‘… for the diagnosis, the remedy, the treatment, the examination I think across the board it would affect how we treat.’ (code 67.9)

Homoeopathic philosophies, such as individualisation of remedy choice, rely heavily on obtaining detailed subjective information from the patient. Participants described how with the lack of guiding symptoms that aid the student and supervising clinician in choosing the individual remedy, the resultant remedy choice may not be accurate:

‘I do think that in that way it sort of compromises our philosophy and what we strive to offer.’ (code 18.3)

Homoeopathic consultations are longer than the average conventional consultation because of the amount of detail required by the student. Participants felt that language barriers negatively affect the time taken to conduct a homoeopathic consultation making it longer than necessary because of the time taken to try to elicit and explain information or shorter than needed because of difficulties in obtaining information:

‘The problem with the language comes in more, I suppose, with the time aspect of things. It takes you longer to get the information you need.’ (code 96.9)

‘I spent almost 20 minutes asking almost the same question.’ (code 96.6)

Informed consent is an incredibly important concept that any health worker needs to uphold. In the context of language barriers, it is difficult to definitively ensure that the health service process is understood by the patient once it has been explained. This raises questions about the validity of the patient’s consent in such scenarios:

‘Well because you’re not able to explain to your patient what you’re going to and when you’re going to do it, why you’re going to do it, what you’re expecting or anticipating, and to check with them if that’s okay. And to know that they fully comprehend what you’re saying and to expect …’ (code 16.4)

‘Oh, with the issues of consent also. It’s actually quite, having to explain to them, but they don’t really understand what you’re saying, but they consent to it anyway.’ (code 16.8)

Language barriers have been found to cause patients to give consent without fully understanding what they are giving consent for because they trust the doctor to make the correct decisions and they are afraid to say ‘no’ in case the HCP abandons their care (Deumert 2010; Levin 2006; Schlemmer & Mash 2006).

### Theme four: The mitigation of language barriers through various strategies

Participants employ various strategies to try and help them to mitigate the effect that language barriers may have on their process and aid in better communication with their patients.

The biggest strategy used by participants is the use of interpreters. Participants described the benefits and challenges of having both informal (those who have not signed formal agreements to interpret for the consultation) and formal (those who are employed to interpret during consultations). Although the participants cannot understand what the interpreter is relaying to the patient, they described a level of mistrust towards the interpreters. The biggest cited reason for this is the disparity in the time it takes for the patient to relay information to the interpreter compared with the time it takes for the interpreter to relay that information back to the practitioner and vice versa:

‘I don’t know if they’re giving what I’m asking in full or if they’re giving what the patient’s stating in full.’ (code 36.5)

‘The translator would have a 5-minute discussion with the patient and only answer you with a one-liner.’ (code 34.10)

HCPs in other studies also experienced an instinctive feeling regarding the inaccuracy of the interpretation and described how they are able to pick up discrepancies from the consultation length and appropriateness of the responses from the patients in response to the questions asked (Deumert 2010; Zendedel et al. 2016).

McCarthy et al. (2013) showed that HCPs appreciate the objective approach that professional interpreters are able to provide to the consultation, providing the practitioner with the relevant information they need to know in order to treat the patient. For similar reasons, the participants felt most comfortable having another student interpret for them, despite the fact that these students are not professional interpreters. Participants felt that student interpreters are more familiar with the medical process and therefore are more likely to relay correct information and obtain the required answers from the patients:

‘And they know how important it is to deliver word for word for what the other person said, I assume.’ (code 30.1)

Participants described how maintaining confidentiality and privacy, and the rights of every patient, is difficult whilst using an interpreter. Particularly if the interpreter has not signed any legal documents ensuring that the information they learn will be kept confidential and private. Directly infringing the right to confidentiality by using informal interpreters is a concern highlighted by participants:

‘I also think it kind of invades their privacy as well. Because now they need to relay this private information, not only to the doctor, but to the translator as well.’ (code 37.10)

‘… it’s a little bit unethical because I don’t think that they’re signing anything to say that they are not going to be telling anybody what they’re hearing in the consult.’ (code 24.5)

There is an ethical and legal responsibility for the HCP to ensure that the fundamental right to privacy is upheld and confidentiality is respected at all times (Demirsoy & Kirimlioglu 2016; McQuoid-Mason 2020; Nell 2006).

### Language acquisition and responsibility to acquire a language

Participants felt that if they were able to acquire the language of the patients, even a few basic phrases, it would eliminate much of the language barrier present. This would have the added benefit of making the patient feel comfortable and building rapport. This may occur even when the HCP is unable to speak the patient’s language yet makes an effort to communicate in their language (Levin 2006; Paternotte et al. 2015). Studies by Deumert (2010), Levin (2011) and Van den Berg (2016) proposed that HCPs learn a new language as a strategy to mitigate language barriers, whilst a study by Levin (2011) showed successful results when practitioners were able to learn new language skills and apply it to their care of patients.

Despite a few issues cited such as lack of motivation, time and perceived difficulty, participants were open to the idea of a language course being implemented into the syllabus.

### Various other strategies employed in the mitigation of language barriers

Participants described a variety of other strategies they employ during consultations to try and mitigate the language barrier, aid in improving communication and elicit necessary information despite the presence of the language barrier. These include adapting questioning for maximal understanding, simplification of language used, checking in with the patient’s understanding, using words that are known in the patients’ language (called code-switching), using visual aides and cues, and exercising patience. When participants feel that they are not going to be able to assist this patient to the best of their ability because of the presence of the language barrier they reported that they would refer to a more appropriate practitioner.

There are studies that show these various strategies: checking in, code-switching, visual aids, gestures – being employed by HCPs in order to mitigate the language barriers they encounter with their patients to varying levels of success (Benjamin et al. 2016; Lukoschek et al. 2003; Sobane & Anthonissen 2013).

## Limitations

The interviews were conducted in English, the researcher’s first language, despite 50% of the sample indicating English as an additional language.

## Recommendations

Language barriers influence the health services that are provided to patients. As the most powerful way of mitigating language barriers is through language, it is recommended that the UJ Faculty of Health Sciences and Department of Complementary Medicine consider the introduction of a basic language course for languages, such as isiZulu, into the syllabus.

As South Africa is a multilingual society, any HCP will likely encounter language barriers with their patients. Within this context, the introduction of South African languages as part of the training of any HCP when aimed at expanding the range of languages spoken by individual students, particularly within a medical context, is highly recommended. This intervention would contribute to a more efficient and equitable healthcare environment by directly contributing to more empowered patients and improved health outcomes arising from less frustration and greater understanding in the consultation environment by both the HCP and patient.

Similar future studies should employ the help of an interpreter to assist during the interview process so as to alleviate any difficulties between the interviewer and participant.

## Conclusion

Participants described language barriers between themselves and their patients as challenging to the development of a relationship of mutual understanding. This lack of relationship may compromise rapport between the patient and the HCP and instils negative emotions such as frustration, inadequacy and insecurity for the HCP. Various aspects of the health services (including case taking and physical examinations) are negatively influenced by language barriers, making these aspects less accurate and more difficult. To cope with such challenges associated with language barriers, students employ various strategies, including the use of interpreters, adaptation of questioning, simplification of language, frequent ‘check-ins’ with the patient, code-switching, visual aides and cues, patience, abandoning inquiry and referral.

Language acquisition and awareness modules introduced early on in the syllabus may be a solution to the mitigation of language barriers and their subsequent challenges. Despite any inherent implications to the curriculum, such initiatives would align with existing language proficiencies of patients who are intended to be assisted. The acquisition of additional languages was felt by participants to be something that would be beneficial to both themselves and their patients.

## References

[CIT0001] Ahmed, S., Lee, S., Shommu, N., Rumana, N. & Turin, T., 2017, ‘Experiences of communication barriers between physicians and immigrant patients : A systematic review and thematic synthesis’, *Patient Experience Journal* 4(1), 122–140. viewed 06 February 2018, from http://pxjournal.org/journal/vol4/iss1/13.

[CIT0002] Almutairi, K., 2015, ‘Culture and language differences as a barrier to provision of quality care by the health workforce in Saudi Arabia’, *Saudi Medical Journal* 36(4), 425–431. 10.15537/smj.2015.4.10133 25828278 PMC4404475

[CIT0003] Ashkinazy, B., 2017, ‘Cultural competence in theory and practice’, Thesis, Georgia State University, viewed 06 February 2018, from https://scholarworks.gsu.edu/anthro_theses/129.

[CIT0004] Benjamin, E., Swartz, L., Hering, L. & Chiliza, B., 2016, ‘Language barriers in health: Lessons from the experiences of trained interpreters working in public sector hospitals in the Western Cape’, *South African Health Review* 2016(1), 73–81, viewed 05 February 2018, from http://hdl.handle.net/10520/EJC189317.

[CIT0005] Bricki, N. & Green, J., 2007, ‘A Guide to using qualitative research methodology’, *Medecins Sans Frontieres* 66(7), 11–13. 10.1109/PROC.1978.11033

[CIT0006] Demirsoy, N. & Kirimlioglu, N., 2016, ‘Protection of privacy and confidentiality as a patient right: Physicians’ and nurses’ viewpoints’, *Biomedical Research* 27(4), 1437–1448. viewed 12 October 2020, from https://www.alliedacademies.org/articles/protection-of-privacy-and-confidentiality-as-a-patient-right-physicians-and-nurses-viewpoints.html.

[CIT0007] DeMotts, O., 2018, *What is trustworthiness in qualitative research?*, viewed 02 August 2018, from https://www.statisticssolutions.com/what-is-trustworthiness-in-qualitative-research/.

[CIT0008] Deumert, A., 2010, ‘“It would be nice if they could give us more language” – Serving South Africa’s multilingual patient base’, *Social Science and Medicine* 71(1), 53–61. 10.1016/j.socscimed.2010.03.036 20452713

[CIT0009] Engelbrecht, C., Nkosi, Z., Wentzel, L. & Govender, S., 2008, ‘Nursing students’ use of language in communicating with isiZulu speaking clients in clinical settings in’, *South African Journal of African Languages* 28(2), 145–156. 10.1080/02572117.2008.10587310

[CIT0010] Eyles, C., Leydon, G. & Brien, S., 2012, ‘Forming connections in the homeopathic consultation’, *Patient Education and Counseling* 89(2012), 501–506. viewed 23 April 2018, from 10.1016/j.pec.2012.02.004 22370197

[CIT0011] Fawole, A., 2014, *Communication strategies of english-speaking foreign medical doctors in the Limpopo province (Thesis)*, University of Limpopo, Limpopo, viewed 08 June 2017, from http://ulspace.ul.ac.za/bitstream/handle/10386/1283/fawole_aa_2014.pdf?sequence=1&isAllowed=y

[CIT0012] Kara, S., 2019, ‘An evaluation of patient profiles and record keeping of the University of Johannesburg Homeopathic health centre in Doornfontein’, MTech, (Homeopathy) [Unpublished], University of Johannesburg, viewed 12 November 2019, from https://ujcontent.uj.ac.za/vital/access/services/Download/uj:31827/SOURCE1?view=true.

[CIT0013] Levin, M., 2006, ‘Language as a barrier to care for Xhosa-speaking patients at a South African paediatric teaching hospital’, *South African Medical Journal* 96(10), 1076–1079. viewed 11 May 2017, from https://www.ncbi.nlm.nih.gov/pubmed/17164939. 17164939

[CIT0014] Levin, M., 2011, ‘Effects on quality of care and healthcare worker satisfaction of language training for healthcare workers in South Africa’, *African Journal of Health Professions Education* 3(1), 11–14, viewed 07 May 2017, from http://www.ajhpe.org.za/index.php/ajhpe/article/view/96.

[CIT0015] Li, C., Abdulkerim, N., Jordan, C. & Ga Eun Son, C., 2017, ‘Overcoming communication barriers to healthcare for culturally and linguistically diverse patients’, *North American Journal of Medicine and Science* 10(3), 103–109. 10.7156/najms.2017.1003103

[CIT0016] Lukoschek, P., Fazzari, M. & Marantz, P., 2003, ‘Patient and physician factors predict patients’ comprehension of health information’, *Patient Education and Counseling* 50(1), 201–210. viewed 23 April 2018, from https://www.ncbi.nlm.nih.gov/pubmed/12781935. 12781935 10.1016/s0738-3991(02)00128-3

[CIT0017] McCarthy, J., Cassidy, I., Graham, M. & Tuohy, D., 2013, ‘Conversations through barriers of language and interpretation’, *British Journal of Nursing* 22(6), 335–339. 10.12968/bjon.2013.22.6.335 23901452

[CIT0018] McQuoid-Mason, D., 2020, ‘COVID-19 and patient-doctor confidentiality’, *South African Medical Journal* 110(6), 461–462. 10.7196/SAMJ.2020.v110i6.14797 32880552

[CIT0019] Nell, J., 2006, *As pects of confidentiality in medical law (Dissertation)*, University of Pretoria, Pretoria, viewed 24 October 2018, from https://repository.up.ac.za/bitstream/handle/2263/26885/00dissertation.pdf?sequence=1

[CIT0020] Parsons, J., Baker, N., Smith-Gorvie, T. & Hudak, P., 2014, ‘To “Get by” or “get help”? A qualitative study of physicians’ challenges and dilemmas when patients have limited English proficiency’, *BMJ Open* 4(6), 1–10. 10.1136/bmjopen-2013-004613 PMC4054645 24902724

[CIT0021] Paternotte, E., Van Dulmen, S., Van der Lee, N., Scherpbier, A. & Scheele, F., 2015, ‘Factors influencing intercultural doctor-patient communication: A realist review’, *Patient Education and Counseling* 98(4), 420–445. 10.1016/j.pec.2014.11.018 25535014

[CIT0022] Refugee Health Technical Assistance Center, 2011, Interpreters vs. Translators, viewed 09 August 2018, from https://refugeehealthta.org/access-to-care/language-access/interpreters-vs-translators/

[CIT0023] Schlemmer, A. & Mash, B., 2006, ‘The effects of a language barrier in a South African district hospital’, *South African Medical Journal* 96(10), 1084–1087, viewed 30 April 2018, from https://www.ncbi.nlm.nih.gov/pubmed/17164941. 17164941

[CIT0024] Skjeggestad, E., Norvoll, R., Sandal, G. & Gulbrandsen, P., 2017, ‘How do international medical graduates and colleagues perceive and deal with difficulties in everyday collaboration? A qualitative study’, *Scandinavian Journal of Public Health* 45(4), 428–435. 10.1177/1403494817698286 28381112

[CIT0025] Sobane, K. & Anthonissen, C., 2013, ‘Linguistic resources and strategies used in multilingual communication in HIV/AIDS care centres in Lesotho’, *Stellenbosch Papers in Linguistics Plus* 42(1), 263–280. viewed 24 April 2018, from http://hdl.handle.net/10520/EJC148742.

[CIT0026] South Africa, 2003, *National Health Act, 2003, (Act 61 of 2003), as amended*, Government Printer, Pretoria.

[CIT0027] Statistics South Africa, 2012, *Census 2011 census in brief*, Statistics South Africa, Pretoria, p. 22.

[CIT0028] Taylor, S., Nicolle, C. & Maguire, M., 2013, ‘Cross-cultural communication barriers in healthcare’, *Nursing Standard* 27(31), 35–43, viewed 03 June 2018, from https://www.ncbi.nlm.nih.gov/pubmed/23641636. 10.7748/ns2013.04.27.31.35.e7040 23641636

[CIT0029] Van den Berg, V.L., 2016, ‘Still lost in translation: Language barriers in South African healthcare remain’, *South African Family Practice* 58(6), 229–231. 10.1080/20786190.2016.1223795

[CIT0030] Verghese, A. & Ioannidis, J., ca. 2017, *The importance of the physical exam*, viewed 25 April 2018, from https://stanfordhealthcare.org/health-care-professionals/medical-staff/medstaff-update/2016-april/importance-physical-exam.html.

[CIT0031] Zendedel, R., Schouten, B., Van Weert, J. & Van den Putte, B., 2016, ‘Informal interpreting in general practice: Comparing the perspectives of general practitioners, migrant patients and family interpreters’, *Patient Education and Counseling, Elsevier Ireland Ltd* 99(6), 981–987. 10.1016/j.pec.2015.12.021 26792389

